# Fault Prediction and Early-Detection in Large PV Power Plants Based on Self-Organizing Maps

**DOI:** 10.3390/s21051687

**Published:** 2021-03-01

**Authors:** Alessandro Betti, Mauro Tucci, Emanuele Crisostomi, Antonio Piazzi, Sami Barmada, Dimitri Thomopulos

**Affiliations:** 1 i-EM S.r.l. (Intelligence in Energy Management), 57121 Livorno, Italy; alessandro.betti@i-em.eu (A.B.); antonio.piazzi@i-em.eu (A.P.); 2 Department of Energy, Systems, Territory and Construction Engineering (DESTEC), University of Pisa, 56122 Pisa, Italy; sami.barmada@unipi.it (S.B.); dimitri.thomopulos@unipi.it (D.T.)

**Keywords:** PV plants, self-organizing maps, fault prediction, inverter module, key performance indicator, lost production

## Abstract

In this paper, a novel and flexible solution for fault prediction based on data collected from Supervisory Control and Data Acquisition (SCADA) system is presented. Generic fault/status prediction is offered by means of a data driven approach based on a self-organizing map (SOM) and the definition of an original Key Performance Indicator (KPI). The model has been assessed on a park of three photovoltaic (PV) plants with installed capacity up to 10 MW, and on more than sixty inverter modules of three different technology brands. The results indicate that the proposed method is effective in predicting incipient generic faults in average up to 7 days in advance with true positives rate up to 95%. The model is easily deployable for on-line monitoring of anomalies on new PV plants and technologies, requiring only the availability of historical SCADA data, fault taxonomy and inverter electrical datasheet.

## 1. Introduction

### 1.1. Motivation

The implementation of accurate and systematic preventive maintenance strategies is emerging nowadays as an essential tool to maintain high technical and economic performance of solar photovoltaic (PV) plants over time [1]. Analytical monitoring systems have been installed worldwide to timely detect possible malfunctions through the assessment of PV system performance [2,3,4,5,6,7,8,9,10]. Due to the abundance of relevant data, and the difficulty in modeling many complex aspects of PV plants, statistical methods based on data mining and machine learning algorithms are recently emerging as a very promising approach both for fault prediction and early detection. In particular, the recent development of key enabling technologies and paradigms, most notably Internet-of-Things (IoT)-environments and machine learning algorithms to handle massive quantities of data, have been recently applied to monitoring the functioning of PV systems. A large number of scientific papers have been written to describe how they can be effectively used to timely detect possible malfunctions through the assessment of an indicator performance, and significant works on the topic include papers [11,12,13,14,15,16,17,18,19]. Besides, similar strategies have been also presented in works that tackle wind farms, see [20,21] with the objective of identifying equipment level failures, while in this case fewer works can be found for the counterpart for PV plants [22].

### 1.2. Paper Contribution

Such an abundance of scientific papers witnesses the interest of the scientific community on this research topic, and the practical importance of developing continuous monitoring algorithms. Indeed, in addition to high customization costs and the need of collecting and transmitting a large number of physical variables, there is a significant interest in developing automatic, non-supervised and accurate methodologies to perform such maintenance strategies, and this is the focus of the present paper. Furthermore, with respect to the aforementioned references, our work does not focus on small-size, usually roof-top-located, PV panels of few kW power, but rather on large-size PV plants that provide power at the scale of several MWs. Most importantly, differently from the previous strategies, we shall show how the maintenance strategies proposed here not only manage to identify possible malfunctioning conditions, but are also able to predict incipient faults a few days in advance from when they occur in practice. In particular, this paper describes a novel and flexible solution for inverter level fault prediction based on a data-driven approach. The model ability to predict or to recognize anomalous patterns and faulty operating conditions is here validated off-line for three different PV plants located in Romania and Greece, for a nominal power of up to 10 MW and a testing window of one year. As we show, the proposed approach has been used in the different plants of different sizes and technologies in the same fashion, and in each case it has proven to provide valuable and accurate failure predictions.

## 2. Case Studies and Methods

**Figure 1:**
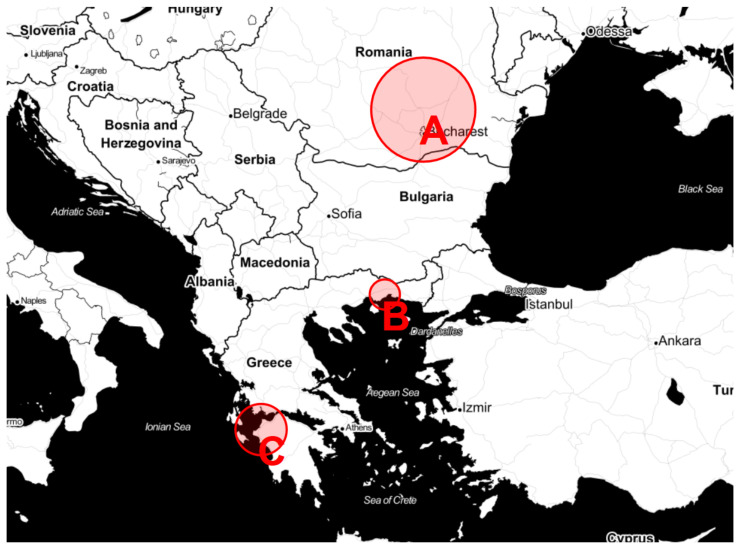
Location of the three considered PV plants, which are denoted as A, B, C. The marker size is proportional to the installed capacity, which is shown in Table 1.

In the paper we shall consider three PV plants, called in the following as plants A, B, and C, respectively, with an installed capacity ranging between 3 and 10 MW, equipped with two different inverter technologies, labelled as 1 and 2, as shown in Table 1. Plant A is located in Romania, whereas plants B and C are in Greece, as shown in Figure 1. Globally, 67 inverter devices have been analysed. More details are given in the following subsections.

### 2.1. PV Plants Details

Plant A is located in Romania and has an installed capacity of around 10 MW, corresponding to 35 inverter modules with a rated output power either of 385 kW AC or 55 kW AC. In the plant both polycristalline and thin film solar panels are installed. The operating facility is able to produce around 15 million kWh per year, corresponding to the annual energy needs of more than 7500 households, thereby avoiding the emission of over 6800 tonnes of $CO_{2}$ into the atmosphere per year.

Plants B and C are located in Greece. Plant B is in the Xanthi region and is composed by strings of thin film solar panels connected to seven inverter modules with a rated ouput power of 385 kW AC, which globally corresponds to an installed capacity of 2.8 MW. On average, it is able to produce about 3.6 million kWh per year. The last considered PV facility is located in the Ilia region and it consists of polycristalline solar panels producing around 6.15 million kWh annually. It is equipped with 25 inverter modules with a rated output power of 183.4 kW AC, which corresponds to an overall installed capacity of 4.9 MW.

### 2.2. SCADA Data and Alarm Logbooks

The datasets of plants A and B consist of 10 measured signals, as explained in Table 2, with a sampling time ν of 5 min. Measured signals include both electrical (DC and AC sides) and environmental quantities (such as the solar irradiance that is acquired by pyranometers, and temperatures). For plant C, also the internal inverter temperature ($T_{int}$) is available. The signals, collected by sensors installed in the PV facility, are stored in a Supervisory Control Furthermore, Data Acquisition (SCADA) system. Data are then transmitted to two higher supervision centers: the Local Control Room of the country of the PV plant, and the World Monitory Room at the headquarters of the power company managing the plant under investigation.

The lengths of the historical datasets are different for the three considered plants, as it is summarized in Table 3, which also shows the number of measured patterns for each dataset. In particular, it is possible to note that the testing period is always at least six months long, and in one case one year long, which is convenient to evaluate the performance of the proposed strategies over different seasons. As it will be described in the greater detail in the following sections, a careful pre-processing stage is required to clean the available data and make them more informative for the training stage.

In addition to the SCADA data, we also exploit the availability of Operation and Maintenance (O&M) logs. In this case, the relevant information consists of the start and end times at which single failure events have been experienced, as well as the specific fault type, and inverter device suffering the failure. Depending on the plant, this information was available either through automatic SCADA logbooks, or through manual logbooks where plant operators manually provided the relevant information. Accordingly, in our model we have also considered the O&M logs, together with the fault taxonomy which is required to associate the manufacturer code with the corresponding failure type, description and severity, which have been used a posteriori to assess the performance of the proposed strategies. In particular, the logbook has been embedded in our model by matching the fault classes listed in the fault taxonomy file to the fault occurrences recorded in the logbooks and associating them with the timestamp of SCADA data. More specifically, a fault of the *k*th type is assigned to timestamp $t_{n}$ if the following condition occurs:

$$t_{start,k}\;\leq \;t_{n}\;\leq t_{end,k}$$

where $t_{start,k}$ ($t_{end,k}$) are the initial (final) instant of the fault event. Once the O&M logs have been discretized consistently with the SCADA data time line, each timestamp $t_{n}$ has been labelled according to the fault code occurring in that instant. Simultaneous fault events at instant $t_{n}$ have been handled according to a prioritization rule, i.e., by labelling $t_{n}$ with the most severe fault code occurring at that instant and, if necessary, the most frequent fault in the day D, with $t_{n}\in D$. As a consequence, the resulting time line is labelled with an integer number, one for each timestamp, corresponding to nominal behaviours (label equal to 0) or faulty events (label larger than 0). While in principle, the information of the specific occurred fault was available, yet in this work we only focus on a binary classification problem where the objective is to discriminate faulty and correct working conditions. Thus, we have trained our algorithms to recognize faulty working conditions, and not the specific type of occurred failure.

### 2.3. Data Pre-Processing

Due to the heterogeneity of the considered physical quantities, the pre-processing stage has been customized specifically for each tag. In particular, the AC power ($P_{AC}$) depends mainly on the solar irradiance (GTI) striking on the PV panel plane and on the environmental temperature ($T_{amb}$). Statistical outliers corresponding to values of $P_{AC}$ significantly larger than 0 despite low values of the GTI, or viceversa, have been removed by implementing a first-order regression of the unknown underlying function $P_{AC}=P_{AC}\left(GTI\right)$ and removing instances that lied far from a linear approximation:

$$\frac{|P_{AC}-\left(GTI\cdot m+b\right)|}{GTI\cdot m+b}>\eta ,$$

where *m* and *b* are the slope and the intercept, respectively, of the linear approximation computed by means of a least-squares fitting, and η is the threshold set by a trial and error process, to identify unrealistically far samples. In addition, many signals exhibit a significant number of not regular data, such as missing or “frozen” samples (i.e., instances where the signal measured by the sensor does not change in time), or values out of physical or operative limits, or spikes. Accordingly, a classic procedure of data cleaning has been carried out to avoid training the algorithms with obviously wrong data. In particular, as many electrical and environmental signals exhibit daily patterns, days having a large percentage of missing data have been removed as a whole.

### 2.4. SCADA Imputation

Since the model, once deployed in practice on-site, must be obviously able to work also in situations of missing online instances, in a “best-effort” fashion (i.e., as well as possible given the obvious difficulties of wrong measurements or wrong data transmission), missing test samples have been imputed according to the classical *k*-Nearest Neighbors (k-NN) algorithm. More in detail, the training set has been used as the reference dataset, replacing missing data with the nearest neighbors according to the Euclidean distance [23,24].

### 2.5. Data Detrending and Scaling

As different electrical (e.g., $P_{AC}$) and environmental (e.g., GTI) signals exhibit seasonal trends, it is convenient to remove such seasonality trends to prevent biased predictions from occurring. In order to remove the season-dependent variability from input data, a detrending procedure has been applied by following tailored approaches for each variable. In particular, the training data of $T_{mod}$ have been deseasonalized by means of the least-squares fitting method to infer the best line $T_{fit}$ against $T_{amb}$ and selecting only low samples with low GTI to remove the effect of the panel heating due to sunlight:

$${\tilde{T}}_{mod}={\left.\frac{T_{mod}-T_{fit}}{T_{fit}}\right|}_{GTI\leq GTI_{thr}},$$

where

$$T_{fit}=m_{T}\cdot T_{amb}+b_{T}$$

is the fitting temperature, $m_{T}$ is the regression slope, $b_{T}$ is the intercept and $GTI_{thr}=100$ W/m${}^{2}$ is a heuristically determined threshold for the solar irradiance to identify “low values of the GTIs” that do not give rise to relevant panel heatings effects (it corresponds to the maximum value of irradiance which does not involve appreciable heating of the module with respect to the ambient temperature).

All the remaining input variables, apart from DC and AC voltages, have been de-trended according to a classical Moving Average (MA) smoothing method to compute the seasonal trend component and applying an additive model for time series decomposition [25,26].

Finally, input data normalization is performed to avoid unbalance between heterogeneous quantities. In particular, we used the standard normalization [26].

## 3. Methodology

The proposed approach consists in training a self-organizing map (SOM) [27,28] neural network with the aim to create a model of the nominal behaviour of the system. For this scope we use an historical dataset, that we denote as training dataset, containing only nominal observations, where faulty instances have been removed. The motivation under this choice is that, as is commonly the case in monitoring applications, most of the measured data correspond to nominal behaviours, and very few cases of faulty patterns are usually measured. The usage of supervised learning methodologies, such as Feed-Forward Neural Networks or Support Vector Machine methods, is not advisable in the case of strongly unbalanced distributions of correct and faulty patterns. On the other hand, unsupervised learning methods are more suitable to represent the structure and the distribution of nominal data. Among unsupervised learning methods, that include clustering and vector quantization algorithms, SOMs are very convenient as they operate a map from the original multi-dimensional space to a two-dimensional space preserving the same topology of the original data (i.e., points that were close to each other in the input space correspond to cells that are still close to each other in the two-dimensional output space). Accordingly, SOMs are an excellent candidate when it is necessary to provide an accurate model of a multivariate distribution of data, and the nonlinear map towards the output space allows us to introduce a number of very useful tools for data analysis, such as the measurement of cell occupancy that has been proposed in this work.

In fact, SOMs have been widely used for condition monitoring applications in other contexts [29,30]. In this manuscript, an original KPI based on the frequency of cells occupancy has been introduced on purpose for our specific application of interest.

In particular, the trained SOM is used to calculate a parameter for each cell of the map, denoted as probability of cell occupancy, which represents the number of training points that are mapped to that particular cell, normalized with respect to the total number of points. During the monitoring stage, new state observations are presented to the SOM and are classified as “in control" or “out-of-control". For this purpose, we calculate the probability of cell occupancy for all the instances measured during the last 24 h, and we compare it against the previously computed probability of cell occupancy. The procedure is now illustrated in more detail.

### Self-Organizing Map Neural Network Based Key Performance Indicator: Monitoring of Cell Occupancy

The SOM output space consists of a fixed and ordered bi-dimensional grid of cells, identified by an index in the range 1,…,D, where a distance metric d(c,i) between any two cells of index *c* and *i* is defined [27]. Each cell of index *i* is associated with a model vector $m_{i}\in R^{1\times n}$ that lies in the same high-dimensional space of the input patterns r∈Δ, where the matrix $\Delta \in R^{N\times n}$ represents the training dataset to be analysed, containing *N* observations of row vectors $r\in R^{1\times n}$. After the training, the distribution of the model vectors resembles the distribution of the input data, with the additional feature of preserving the grid topology: model vectors that correspond to neighbouring cells shall be neighbours in the high-dimensional input space as well.

When a new input sample r is presented to the network, the SOM finds the best matching unit (BMU) *c*, whose model vector $m_{c}$ has the minimum Euclidean distance from r:

$$c={\mathrm{argmin}}_{i}\{∥r-m_{i}∥\}.$$

In this case we say that the input pattern r is mapped to the cell *c*. In order to assess the condition of newly observed state patterns to be monitored, we introduce the following KPI:

(1) $$KPI\left(d\right)=\sum_{i=1}^{D}p_{i,d}\frac{1-|p_{i,TRAIN}-p_{i,d}|}{1+|p_{i,TRAIN}-p_{i,d}|}$$

where *d* denotes a test day index, and the probability of cell occupancy during day *d* is defined as

$$p_{i,d}=\frac{N_{i,d}}{N_{d}},i=1\ldots D,$$

where $N_{d}=24\cdot 60/\nu$ is the total number of samples in a day, and $N_{i,d}$ is the number of samples, within day *d*, that were mapped to cell *i*. In the same fashion the probability of cell occupancy in the training phase is defined as

$$p_{i,TRAIN}=\frac{N_{i,TRAIN}}{N},i=1\ldots D$$

where $N_{i,TRAIN}$ represents the number of training patterns that were mapped to cell *i*, while *N* is the total number of training samples. It is straightforward that

$$0\leq p_{i,d}\leq 1,\forall i=1\ldots D$$

and

$$0\leq p_{i,TRAIN}\leq 1,\forall i=1\ldots D.$$

As a result, the KPI(d) value defined in Equation (1) is calculated once a day, based on the analysis of the measurements of the previous 24 h.

If the test samples of the day *d* being monitored represent mainly nominal observations, then the corresponding $p_{i,d}$ values shall be close to $p_{i,TRAIN}$ values, that were calculated using nominal historical observations. In this case the resulting value of the KPI in Equation (1) tends to 1. Conversely, if the patterns of day *d* contain abnormal conditions, then the cell occupancy will be mainly altered, leading to a situation where for a significant number of cells $|p_{i,TRAIN}-p_{i,d}|$ tends to 1 ($p_{i,d}$ close to 1 and $p_{i,TRAIN}$ close to 0 or viceversa). In this case the resulting KPI value tends to 0.

From a physical point of view, the proposed KPI is a robust indicator that is able to detect changes in the underlying non-linear dynamics of the plant. The normal status is represented by KPI=1, while decreasing values represent a deviation from healthy conditions.

In particular, we have accurately tuned a set of rules and thresholds based on the KPI values, in order to generate warning levels of different severity, as summarized in Table 4. The following two thresholds are defined as lower control limits:

$$thr_{1}=\mu -3\sigma ,$$

and

$$thr_{2}=\mu -5\sigma ,$$

where μ and σ represent, respectively, the mean value and the standard deviation of the *KPI* values calculated as in Equation (1) using all the training patterns.

The logic of the generation of the warnings takes into account the crossing of the thresholds, the persistence of the KPI values below the thresholds and the derivative of the KPI. In particular, a negative derivative, representing a progressive degradation of the health state of the plant, is a necessary condition for the generation of a warning. This choice has the effect to avoid the generation of warnings during the positive derivative of KPI, that usually correspond to a period where the plant is gradually returning to a normal state after a maintenance intervention. In this way the number of false positives is greatly reduced.

## 4. Results and Discussion

**Figure 2:**
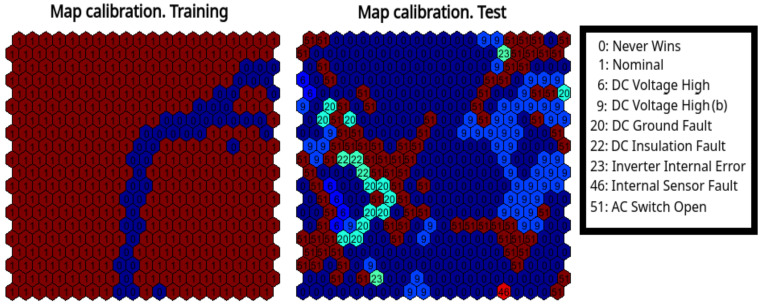
Calibration of the trained SOM, using training data and test data.

The proposed model has been trained on the training set as specified in Table 3, and in this section we discuss the outcome of the testing stage. A 20×20 map was used (D=400), with hexagonal cells int the topological space, and the sequential update rule was used to train the map [27]. Figure 2 shows the a-posteriori calibration of the trained map for the inverter A.2 of plant A. Each cell of the calibration map is associated with a label related to the fault class of the patterns that were mapped to that cell most times. Obviously, in the training calibration map only the nominal class is present, and the obtained map results to be divided in two clusters related to day (left side) and night (rigth side). The test calibration map was created using only faulty patterns, yielding the distribution shown in the right side of Figure 2, where it can be noticed that different kind of faults are mapped to nearby cells related to day and night regions.

As a result, our system was able to identify a significant amount of failure events, which we could validate using the available data, and a selection of the most interesting ones is discussed in more detail in this section.

### 4.1. Plant A

Table 5 lists the most relevant faults occurred on the inverter module A.2 of plant A in the test period 01 October 2014 to 30 September 2015, i.e., 1 year long. For each failure, the table reports the specific fault, the time interval until the problem was fixed, and its severity in a scale from 1 (most critical) to 5 (least critical), as defined by the inverter manufacturer. According to the alarm logbook, this plant experienced a number of thermal issues on several different devices which lead to a non-correct heat dissipation. Such damages led to a production loss estimated in some thousands of euros and required the replacement of many components of the inverter of plant A in August–September 2015.

**Figure 3:**
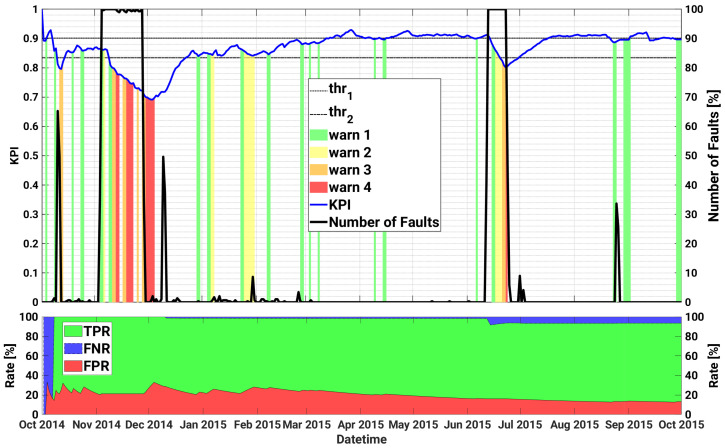
Historical case studies for inverter A.2 of plant A. Top plot—Left axis: KPI, as well as the warning levels and the upper and lower thresholds are shown as a function of datetime; Right axis: time series of daily number of faults. Bottom plot: TPR, FNR and FPR as a function of datetime.

Figure 3 illustrates, on the top part, the curve of the proposed daily KPI (in blue), as well as the warning levels triggered by the KPI, with different colours depending on the severity of the warning, ranging from green (warning level 1—least critical), to red (warning level 4—the most critical). In order to evaluate the ability of the proposed KPI to detect anomalous working conditions, we also show in black the normalized number of the true faulty instances ${\hat{N}}_{fault}$ that were registered on each day. In particular, the normalized number of true faulty instances on the d−th day is computed as:

(2) $${\hat{N}}_{fault}\left(d\right)=\frac{\mathrm{number}\;\mathrm{of}\;\mathrm{faulty}\;\mathrm{instances}\;\mathrm{in}\;\mathrm{day}\;d-1}{N_{d}}\cdot 100\%,$$

Roughly speaking, Equation (2) may give rise to value between 0, i.e., no fault observed in the day, up to 100%, indicating abnormal days with all the $N_{d}$ instances labelled as faulty. The two thresholds $thr_{1}$ and $thr_{2}$ are also represented by dashed and dotted black curves, respectively.

Additionally, in order to make a quantitative performance evaluation, in the bottom of Figure 3, it is possible to observe the True Positive Rate (TPR), the False Negative Rate (FNR), and the False Positive Rate (FPR), as a function of the date. In particular, the TPR is defined as the ratio of the true positive (faults) with respect to the actual faults (i.e., TPR = TP/P); the FNR is defined as the ratio of the false negatives (i.e., faults that were not recognized as faults) with respect to the actual faults, so that FNR = FN/P; and FPR, which is defined as the ratio of the False Positives with respect to the non-faults cases (i.e., it corresponds to the rate of false alarms). In practice, a faulty sample has been classified as a TP if at least one warning is triggered in the previous 7 days, otherwise it is classified as FN. On the other hand, if an alert is raised and no faults occur in the following week, the corresponding sample is taken as a FP. As can be seen, a clear correlation between the warning alerts and the actual faults is observed, with the most severe warnings triggered in correspondence of the most critical days, i.e., those having a higher percentage of registered faulty instances. Additionally, although the daily generated KPI may introduce a delay in the generation of predictive alerts, it is effective in minimizing the amount of generated false positives and false negatives instances, thanks also to the monitoring of the trend derivative.

**Figure 4:**
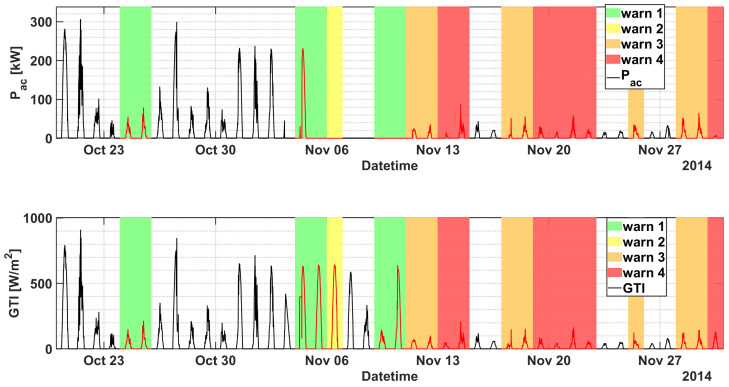
$P_{ac}$ of inverter A.2 (top) and GTI (bottom) as a function of datetime in the period 20 October 2014–30 November 2014 (plant A). The warning levels are superimposed for convenience.

**Figure 5:**
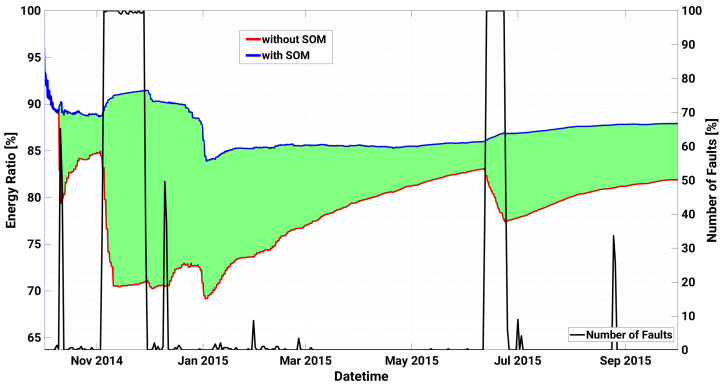
Left axis: energy ratio with respect to the ideal case with and without the application of the SOM based model for inverter A.2 of Plant A. The green area represents the maximum energy gain achievable by enabling it. Right axis: time series of daily number of faults.

In particular, the first critical failure (AC Switch Open), that gives rise to almost 60% of device faults in a day, is observed on 10 October 2014. The model anticipates the failure triggering warnings of level 1 on both October 4 and on October 9, with a significant degradation of the KPI in correspondence of the fault. The same failure occurs again on 3 November 2014 for a more prolonged number of days (until 28 November 2014), and for 26 consecutive days the SCADA registers almost 100% of daily faults of the device, and almost no power generation at all. The SOM early detects the anomaly with a remarkable drop of the KPI from 4 November, triggered by an unexpected zero power generation for an almost fully sunny day, as shown in Figure 4. However the first sign of abnormal behaviour had been predicted almost 10 days before with warnings of level 1 occurring on the 24 and 25 October, and with the KPI well below the first warning threshold $thr_{1}$. Furthermore, during the prolonged fault, the KPI notifies the operators with a degree of criticality that progressively increases up to the maximum level 4, thus strongly advising the plant operator to proceed with the reactive maintenance action. In particular, as can be seen in Figure 5, application of the proposed method and timely maintenance interventions could have led to an energy gain up to roughly 20%.

Conversely, it is interesting to note that the proposed method does not trigger any alert in correspondence of the DC Insulation fault, i.e., an overvoltage across the DC capacitors, that occurs on 9 December, due to the positive value of the KPI derivative. However, the last warning of level 4 activated on 4 December would have allowed the O&M team to plan a maintenance intervention and solve this issue in time. Then, from the beginning of year 2015 the overall trend of the KPI exhibits a slow but almost constant increase, with some alerts up to level 2 that occur in correspondence, or even ahead, of some minor, yet actual, faulty events.

The second most severe failure starts on the 11 June 2015 due to an overvoltage across the bulk capacitors of the DC/DC converter. Remarkably, also in this case the SOM realizes of the anomalous behaviour already on 6 June, and triggers a first warning of level 1. Additionally, a sudden KPI drop is observed in correspondence of the failure, with warnings generated up to level 4. After this failure, the nominal behaviour is restored and the model does not generate any alert until the end of August 2015, when the model predicts an anomaly on 23 August, which is followed by an actual registered fault that occurs the following day.

The performance over the whole test set are remarkable, with a TPR exceeding 93% (FNR < 7%) and a FPR of almost 13%.

### 4.2. Plant B

**Figure 6:**
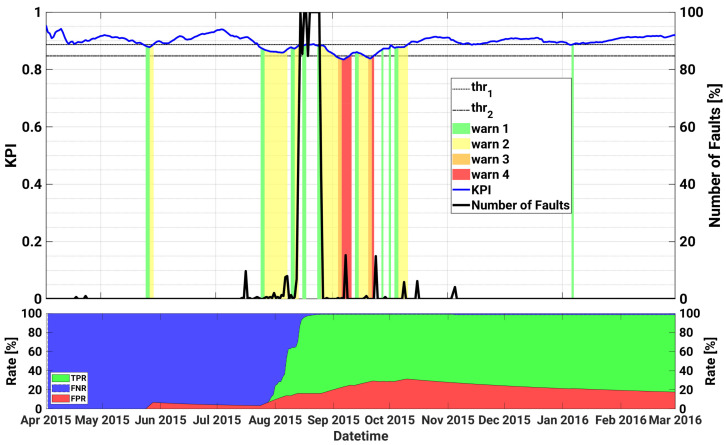
Historical case studies for inverter B.1 of plant B. Top plot—Left axis: KPI, as well as the warning levels and the upper and lower thresholds are shown as a function of datetime; Right axis: time series of daily number of faults. Bottom plot: TPR, FNR and FPR as a function of datetime.

Table 6 lists the most critical failures occurred on inverter B.1 installed in plant B in the test period of interest from 1 April 2015 to 29 February 2016, whereas in Figure 6 the proposed KPI, the warning levels, as well as the daily number of faults are plotted as a function of time for the same device. In the first part, the KPI is almost always above the safety threshold and does not detect the DSP communication error between the inverter internal control devices that occurs on 16 July 2015. After that, the KPI starts to decrease and realizes of an incoming failure on 26 July, generating alerts up to level 2. A real failure occurs indeed on 6 August, due to an internal sensor error in the measurement of the leakage current on the DC side. A consequent maintenance action is then scheduled to verify the issue.

Then a new fault is predicted on August 10, with warnings triggered up to level 2. An overvoltage across the bulk capacitors on the DC side occurs indeed on 13 August, and it lasts almost 13 days, causing also the replacement of the inverter. The warnings triggered by the indicator during the failure are thus explained with the lack of data in these days.

From 24 August, the KPI starts signaling new faulty conditions, with alerts that progressively become more critical, up to level 4, in correspondence of an internal sensor fault that is registered by the system on 7 September. The consequent inspection of the O&M operators confirmed the fault and caused the replacement of a cooling fan in the inverter. Similarly, from 14 September, the KPI starts again progressively to decrease with new warnings that get up to level 4, when a failure is again registered on 23 September. On that day, a new corrective intervention is scheduled which causes the substitution of the inverter cooling pump. Then the KPI recovers safe values, with some alerts generated in correspondence of minor faulty events, with very few missed detections or misdetections.

Furthermore, for the second PV plant, the KPI performs in an accurate fashion, with a TPR exceeding 98% (FNR < 2%) and a FPR equal to about 18%.

### 4.3. Plant C

**Figure 7:**
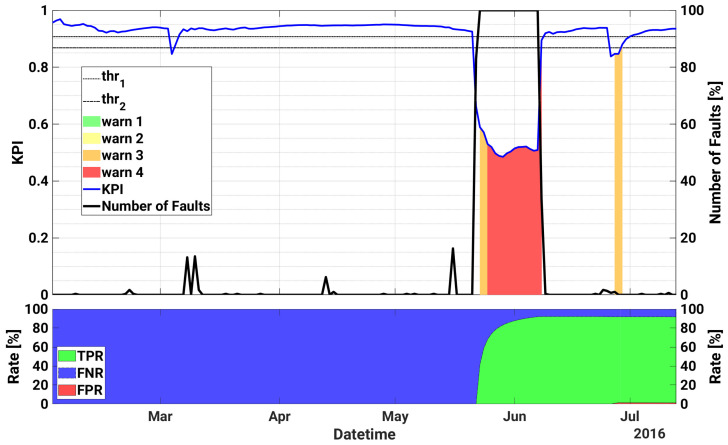
Historical case studies for inverter 3.5 of plant C. Top plot—Left axis: KPI, as well as the warning levels and the upper and lower thresholds are shown as a function of datetime; Right axis: time series of daily number of faults. Bottom plot: TPR, FNR and FPR as a function of time.

Table 7 lists the most severe failures registered for inverter 3.5 of plant C in the testing period, from 1 February to 27 July 2016. As in the previous cases, Figure 7 shows the proposed KPI, the warning levels and the daily number of faults as a function of time for the same module. As can be seen in Table 5, the device does not experience particularly important failures until the last decade of May. Indeed, the registered failures are mainly due either to some parameters outside of the standard values or by scheduled maintenance actions.

**Figure 8:**
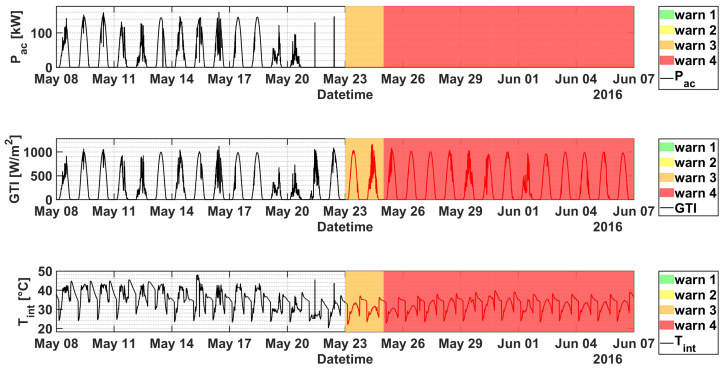
From top to bottom: $P_{ac}$, GTI and $T_{int}$ of inverter 3.5 of plant C as a function of datetime in the period 8 May–7 June 2016. The warning levels are superimposed for convenience.

Accordingly, the model does not detect any relevant issue until 21 May (see Figure 7), when the KPI suddenly drops triggering alerts up to level 4. Looking at the single signals (Figure 8), an obvious anomaly is given by the power generation that is equal to zero irrespectively of the sunny weather conditions. Similarly, an unmotivated drop in the internal inverter temperature occurs. The technical inspection on the plant confirms the issue, which was caused by an IGBT stack fault and led to a production loss estimated roughly in 16 MWh. The whole inverter is then replaced after the failure. Then the KPI comes back to take safe values, generating only some alerts around 27 June, in correspondence of minor grid failures caused by mains parameters out of range.

The KPI works in an accurate way also for plant C, as can be seen in the bottom plot of Figure 7. In fact, the TPR is almost 92% (FNR = 8%) and FPR is just roughly 1%.

## 5. Conclusions

In Table 8, we summarize the performance results of the proposed method obtained in the three case studies. In particular, excellent performances are obtained in terms of TPR values, and good results are also achieved in terms of FPR for all the case studies. The predictive capacity of the proposed method is summarized in Table 9 reporting the dates of the occurrence of the faults, and the dates when such faults had been predicted by the proposed KPI. On average, the KPI predicts incipient faults between 6 and 7 days before they are observed in practice. Furthermore, in addition to being able to predict the faults, the KPI also exhibits excellent early detection capabilities, by signaling with increasing warning levels as the faults evolve and reach more severe conditions.

The proposed SOM-based monitoring system is now being installed in PV plants for online condition monitoring and the preliminary feedback from plant operators is very positive. A full evaluation of the online system will be the subject of our future work. Furthermore, we are currently developing a supervised fault-classification tool that we plan to integrate in the system in order to predict the specific class of fault, in addition to recognizing a generic faulty condition, as in our presented work.

## Figures and Tables

**Table 1:** List of considered PV plants: plant A is located in Romania, whereas plants B and C in Greece.

Plant Name	Number of Inverter Modules	Inverter Manufacturer Number	Max Active Power [kW]	Plant Nominal Power [MW]
A	35	1	385	9.8
B	7	1	385	2.8
C	25	2	183.4	4.9

**Table 2:** List of electrical and enviromental signals used as input tags. $T_{int}$ is only available for plant C.

Signal Number	Signal Type	Signal Name	Variable Name	Unit
1	Electrical	DC Current	$I_{DC}$	[A]
2	Electrical	DC Voltage	$V_{DC}$	[V]
3	Electrical	DC Power	$P_{DC}$	[W]
4	Electrical	AC Current	$I_{AC}$	[A]
5	Electrical	AC Voltage	$V_{AC}$	[V]
6	Electrical	AC Power	$P_{AC}$	[W]
7	Environmental	Internal Inverter Temperature	$T_{int}$	[°C]
8	Environmental	Panel Temperature	$T_{mod}$	[°C]
9	Environmental	Ambient Temperature	$T_{amb}$	[°C]
10	Environmental	Global Tilted Irradiance	GTI	[W/m${}^{2}$]
11	Environmental	Global Horizontal Irradiance	GHI	[W/m${}^{2}$]

**Table 3:** Temporal extension of the data-sets and data used for training and for testing for each PV plant. The number of measured patterns is also shown (patterns are 10 dimensional for plants A and B, and 11 dimensional for plant C).

Plant Name	Training Period (dd/mm/yyyy)	Test Period (dd/mm/yyyy)
A	from 20/03/2014 to 30/09/2014 n° of patterns: 55,872	from 01/10/2014 to 30/09/2015 n° of patterns: 104,832
B	from 27/10/2014 to 31/03/2015 n° of patterns: 44,640	from 01/04/2015 to 29/02/2016 n° of patterns: 96,192
C	from 01/02/2015 to 31/01/2016 n° of patterns: 104,832	from 01/02/2016 to 27/07/2016 n° of patterns: 50,976

**Table 4:** Logic for the generation of the warning levels.

Warning Level	Thresholds	KPI Derivative	Persistence
1	KPI<μ−3σ	<0	1 day
2	KPI<μ−3σ	<0	≥2 days
3	KPI<μ−5σ	<0	1 day
4	KPI<μ−5σ	<0	≥2 days

**Table 5:** Main failures occurred on inverter A.2 of plant A in the historical period.

Fault Name	Severity (1 to 5)	Start Date (dd/mm/yyyy)	End Date (dd/mm/yyyy)
AC Switch Open	2	10/10/2014	11/10/2014
AC Switch Open	2	03/11/2014	28/11/2014
DC Insulation Fault	2	09/12/2014	10/12/2014
DC Voltage High	2	11/06/2015	23/06/2015
AC Switch Open	2	24/08/2015	25/08/2015

**Table 6:** Main failures occurred on inverter B.1 of plant B during the testing period.

Fault Name	Severity (1 to 5)	Start Date (dd/mm/yyyy)	End Date (dd/mm/yyyy)	Notes
Communication Error	2	16/07/2015	16/07/2015	None
Internal sensor fault	2	06/08/2015	07/08/2015	Fault Log downloading
DC Voltage High	2	13/08/2015	25/08/2015	Device B.1 replaced
Internal sensor fault	2	07/09/2015	07/09/2015	Cooling fan replaced
Internal sensor fault	2	23/09/2015	23/09/2015	Cooling pump replaced

**Table 7:** Main failures occurring on inverter 3.5 of plant C during the testing period.

Fault Name	Severity (1 to 5)	Start Date (dd/mm/yyyy)	End Date (dd/mm/yyyy)	Notes
AC Voltage out of range	3	07/03/2016	07/03/2016	Grid fault
AC Voltage out of range	3	09/03/2016	09/03/2016	Grid fault
AC Voltage out of range	3	12/04/2016	12/04/2016	Grid fault
AC Voltage out of range	3	15/05/2016	15/05/2016	Scheduled maintenance
AC Switch Open	2	21/05/2016	07/06/2016	Inverter 3.5 replaced

**Table 8:** Summary of the performance results on the three case studies.

Test Case	TPR	FNR	FPR
Plant A, inv. A.2	93%	7%	13%
Plant B, inv. B.1	98%	2%	18%
Plant C, inv. 3.5	92%	8%	1%

**Table 9:** Summary of the predictive performance of the proposed method.

Test Case	Date of Fault Occurrence (dd/mm/yyyy)	Date of Fault Prediction (dd/mm/yyyy)	Time in Advance of Prediction
Plant A, inv. A.2	10/10/2014	4/10/2014	6 days
Plant A, inv. A.2	3/11/2014	24/10/2014	10 days
Plant A, inv. A.2	09/12/2014	last warning on 04/12/2014	(5 days) fault occurs during plant maintenance
Plant A, inv. A.2	11/06/2015	06/06/2015	5 days
Plant A, inv. A.2	24/08/2015	23/08/2015	1 day
Plant B, inv. B.1	16/07/2015	not detected	- minor fault
Plant B, inv. B.1	06/08/2015	26/07/2015	10 days
Plant B, inv. B.1	13/08/2015	10/08/2015	3 days
Plant B, inv. B.1	07/09/2015	24/08/2015	14 days
Plant B, inv. B.1	23/09/2015	14/09/2015	9 days
Plant C, inv. 3.5	21/05/2016	21/05/2016	0 days
